# SEDA 2024 update: enhancing the SEquence DAtaset builder for seamless integration into automated data analysis pipelines

**DOI:** 10.1186/s12859-024-05818-2

**Published:** 2024-05-27

**Authors:** Miguel Reboiro-Jato, Daniel Pérez-Rodríguez, Miguel José Da Silva, David Vila-Fernández, Cristina P. Vieira, Jorge Vieira, Hugo López-Fernández

**Affiliations:** 1 SING Research Group, SERGAS-UVIGO, Galicia Sur Health Research Institute (IIS Galicia Sur), 36213 Vigo, Spain; 2 CINBIO, Department of Computer Science, ESEI-Escuela Superior de Ingeniería Informática, Universidade de Vigo, 32004 Ourense, Spain; 3 NeuroEpigenetics Lab, Instituto de Investigación Sanitaria de Santiago (IDIS), Complejo Hospitalario Universitario de Santiago, 15706 Santiago de Compostela, Spain; 4 Translational Neuroscience Group, Área Sanitaria de Vigo-Hospital Álvaro Cunqueiro, SERGAS-UVIGO, CIBERSAM-ISCIII, Galicia Sur Health Research Institute (IIS Galicia Sur), 36213 Vigo, Spain; 5 Instituto de Investigação e Inovação em Saúde (I3S), Universidade do Porto, Rua Alfredo Allen, 208, 4200-135 Porto, Portugal; 6 Instituto de Biologia Molecular e Celular (IBMC), Rua Alfredo Allen, 208, 4200-135 Porto, Portugal

**Keywords:** FASTA, Reproducibility, Pipelines, Workflows, Docker

## Abstract

**Background:**

The initial version of SEDA assists life science researchers without programming skills with the preparation of DNA and protein sequence FASTA files for multiple bioinformatics applications. However, the initial version of SEDA lacks a command-line interface for more advanced users and does not allow the creation of automated analysis pipelines.

**Results:**

The present paper discusses the updates of the new SEDA release, including the addition of a complete command-line interface, new functionalities like gene annotation, a framework for automated pipelines, and improved integration in Linux environments.

**Conclusion:**

SEDA is an open-source Java application and can be installed using the different distributions available (https://www.sing-group.org/seda/download.html) as well as through a Docker image (https://hub.docker.com/r/pegi3s/seda). It is released under a GPL-3.0 license, and its source code is publicly accessible on GitHub (https://github.com/sing-group/seda). The software version at the time of submission is archived at Zenodo (version v1.6.0, http://doi.org/10.5281/zenodo.10201605).

**Supplementary Information:**

The online version contains supplementary material available at 10.1186/s12859-024-05818-2.

## Background

We developed SEDA (SEquence DAtaset builder) as a multiplatform desktop application to deal with FASTA files, one of the most used formats to store DNA and protein sequences [1]. The initial version of SEDA was characterized for offering an easy-to-use graphical user interface to a collection of more than thirty operations. These include common operations such as filtering, sorting, and editing. However, unlike other applications for manipulating FASTA files (such as SeqKit [2], seqtk [3] or seqmagick [4]), SEDA also provides advanced operations for performing BLAST queries, protein domain annotation, and gene annotation. For these advanced operations, SEDA uses external software (Splign/Compart [5], Augustus [6], CGA [7]) as well as popular web services (NCBI BLAST [8], UniProt BLAST [9], or PfamScan [10]). While this initial version of SEDA eases the work of life science researchers working with DNA and/or protein sequences—especially those who have no programming skills—, it could not be used in the context of command-line scripts or automated analysis pipelines as it lacks a command-line interface (CLI).

This paper focuses on detailing the enhancements made in the latest release of SEDA, including: (i) the incorporation of a comprehensive CLI for all operations; (ii) the introduction of new features, such as a new gene annotation operation for executing the CGA (Conserved Gene Annotation) pipeline; (iii) the development of a framework for constructing automated pipelines of SEDA commands using Compi [11]; (iv) enhanced integration in Linux environments, with man help and new distributables for APT (Advanced Package Tool), used in Debian-based distributions like Ubuntu or Kubuntu, RPM (Red Hat Package Manager), used in Fedora or CentOS, among others, and Snap, a package manager available across a range of Linux distributions.

## Implementation

SEDA is implemented in Java 8 using the GC4S library for GUI development [12] and a custom framework for CLI development.

The project has a modular architecture with plugins, featuring a central module responsible for core SEDA functionalities, including managing sequences and files, and additional modules offering supplementary operations. As outlined in the developer’s section of the SEDA manual, extending SEDA is straightforward through the incorporation of new plugins, seamlessly integrating new functionalities into the main application.

Some functions within SEDA require the use of external software like BLAST, Clustal Omega, EMBOSS, Splign/Compart, ProSplign/ProCompart or bedtools, among others. Although SEDA distributions for Windows, Linux, and Mac OS allows users to specify the paths for these dependencies if required, it is noteworthy that some of them, such as Splign/Compart and ProSplign/ProCompart, are exclusive to Linux or might pose installation challenges. To streamline SEDA usage and tackle such drawbacks, Docker images corresponding to each dependency have been developed and SEDA can execute third-party software through these images instead of relying on local binaries. Consequently, when executing operations requiring external applications, SEDA only needs Docker, which can be easily installed across the three primary operating systems. This approach aligns with SEDA’s user-centric ethos, eliminating common issues faced by users lacking advanced technical expertise, particularly the installation and configuration of third-party dependencies. Nevertheless, it is important to note that these external dependencies are only required for gene annotation pipelines, BLAST-based operations, and Clustal Omega alignment; the majority of SEDA's operations remain accessible without them.

The “SEDA pipelines with Compi” framework was developed using Compi, a versatile framework for constructing computational pipelines [11].

## Results

### Command line interface

Having a CLI is essential for enabling the use of SEDA in automated environments, whether it is within scripts or as part of automated analysis workflows. The CLI was developed following standard conventions and providing rational default parameters to reduce the complexity of the commands.

As the updated online manual shows [13], each command mimics the GUI configuration as much as possible, taking into account the necessary changes. Full information about each command and its parameters can be obtained with “ < command_name > --help” or using the man pages, as commented later. Additional file 1 provides a list of all the commands and categories and their corresponding GUI operations. Table 1 shows the command options that are common to all commands: input, output, processing configuration, and configuration files (see below). These options are shown in categories in the help of each command, along with an additional section at the beginning with the command specific options. This way, the GUI layout is somehow reproduced in the CLI, facilitating its interactive usage and adoption.

**Table 1:** SEDA options common to all commands

Type	Name	Description
Input	--input-directory/-id	Path to the folder containing the files to process
	--input-file/-if	Path to the file to process. This parameter can be specified multiple times
	--input-list/-il	Plain-text file with the paths of the files to process
Output	--output-directory/-od	Path to the folder to be created where result files will be saved
	--output-group-size/-sz	Whether output files must be split into subdirectories of a defined size. By default (0), no split subdirectories are created. (default: 0)
	--output-gzip/-gz	Whether the output files must be compressed using gzip
Configuration	--in-disk-processing/-dp	Whether files must be processed in hard disk. If not specified, files are processed in RAM memory. This option is slower but allows processing big batches of files with thousands of sequences
Command configuration files	--parameters-file/-pf < parameters-file >	File with the command configuration (created using --save-parameters-file/-spf or the GUI) to load the command options
	--save-parameters-file/-spf	File to save the command configuration options for later reuse

One interesting feature is the interoperability of GUI and CLI operations through JSON configuration files. Additional file 2 provides the JSON configuration files of three different operations. Both the GUI and the CLI allow recording the operations’ configuration in these human-readable files to be reused later. This way, for instance, it is possible to configure (and run) an operation with the GUI and save its state for later use (e.g. to reproduce a series of steps automatically using the CLI). In addition, this feature also helps to ensure the reproducibility of the analyses and can be advantageous when creating pipelines with the procedures described below.

### New functionalities

Since its initial stable release, SEDA has undergone a continuous series of updates, encompassing bug fixes and various enhancements. Among these, a noteworthy addition is the incorporation of a new gene annotation operation designed to execute the CGA pipeline. This addition further strengthens SEDA's unique capability in performing advanced operations.

CGA [7] is a Compi pipeline to efficiently perform CDS annotations by automating the steps that researchers usually follow when performing manual annotations. Within CGA, the following procedure is applied to nucleic input sequences in a given FASTA file with the genome regions of interest. It starts with the “get-orf” step, which identifies open reading frames (ORFs) longer than 30 bp using EMBOSS getorf program. The obtained sequences are then translated in frame + 1 using EMBOSS transeq program, resulting in “01_orfs.prot.fasta” and “01orfs.nuc.fasta files”. Subsequently, the "blast" step creates a BLAST database from the identified ORFs and performs a blastp analysis against the user-provided reference protein sequence. Significant matches (e-value < 0.05) are retrieved and stored in the “02_*.ini” files. The “sort” step organizes the “02_*.ini” files based on the relative genomic locations of exons, producing “03_*.ini.sorted” files. The “join-exons” step iteratively processes the sorted files, applying consecutive sub-steps for merging sequences, extracting splicing sites, translating sequences, removing stop codons, and aligning sequences with the reference protein. This cycle continues until all exons are successfully joined, creating the main output files “04_*.join_exons_results”. The “predict” step processes these files using EMBOSS getorf and transeq to obtain predicted CDS and protein sequences in frame + 1, considering a minimum size specified by the user. Additionally, a blastp search is performed using the reference protein as the query. The result includes four output files for each input sequence: the “05_*.join_exons_results” files containing the DNA sequences being considered before the predict step (useful for manual sequence refinement when there are reasons to believe that a complete annotation was not achieved; the “05_*.nuc” and “05_*.pep” files containing the predicted CDS sequences and their translations, respectively; and the “05_*.pep.blast” files showing the result of the blastp search when using the reference protein as query and the corresponding “05_*.pep” file as the database, that provide a fast and simple way of checking how different the annotated sequences are from the reference protein.

### Pipelines

As commented before, the new CLI allows using SEDA in automated environments and thus it can now be seamlessly integrated in automated analysis workflows or pipelines. For instance, Auto-Phylo is a recently developed pipeline maker software for phylogenetic studies [14], which includes many modules where SEDA CLI operations are used. Such usage enables the development of new modules by users with basic Bash scripting capabilities, and improves code readability [15].

Also, within the first SEDA release, we published three SEDA protocols with step-by-step execution guides for preparing datasets for large-scale phylogenetic analyses, obtaining protein family members, and performing phylogenomic studies. By providing the operations’ configuration files, these protocols could be re-executed manually with updated data. Now, with the introduction of the CLI, these processes can now be fully automated. Since these protocols mainly consist of SEDA operations (with very few exceptions of custom Bash scripts), we automated them by creating ready-to-run pipelines. Nevertheless, instead of creating tailored pipelines, we developed the “SEDA pipelines with Compi” framework, which is publicly available at GitHub [16].

Compi is a versatile framework for constructing computational pipelines. These pipelines are specified in an XML file, containing the pipeline parameters, tasks definitions and task dependencies. Therefore, “SEDA pipelines with Compi” can be seen as a specialization of Compi for SEDA. In this specialization, the definition of the tasks to be executed by SEDA is streamlined, eliminating redundant and routine elements. Additionally, task communication is automated through a convention-based management of tasks inputs and outputs. With this simplification, the creation of a pipeline for SEDA is reduced to the definition of its tasks (i.e. SEDA commands) and their execution order (including dependencies on other tasks).

The choice of Compi as underlying workflow manager technology was motivated by technical issues compared to existing alternatives like Nextflow [17] or Snakemake [18]. For instance, Snakemake follows a rule-based approach to specify data processing pipelines in which each rule describes how to create one or more output files from one or more input files. On the other hand, Nextflow pipeline scripts are created by defining independent processes that communicate to each other via input and output channels. While powerful and widely used in other use cases, these approaches are not appropriate for creating generic workflows as Compi allows thanks to the declarative XML and the possibility of using custom task runners.

**Fig. 1:**
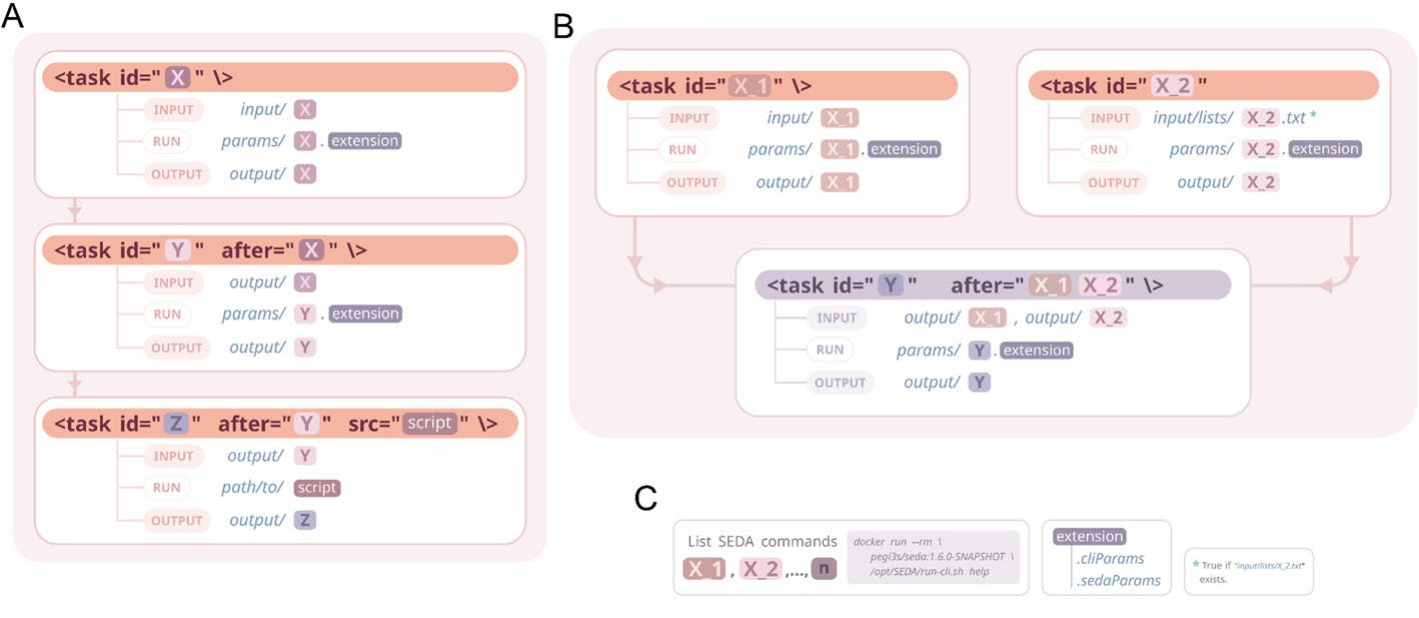
Representation of tasks in a SEDA pipeline

There are some conventions to consider when using "SEDA pipelines with Compi" (illustrated in Fig. 1). First, task ids are the name of the SEDA commands to be executed (X, Y and Z in Fig. 1A). Second, if the same SEDA command is executed more than once, then tasks ids are disambiguated by adding a numeric suffix (X_1 and X_2 in Fig. 1B). Third, the location of the input files of each task depends on whether it has dependencies or not. For tasks without dependencies (i.e. initial tasks) they are located at input/ < seda_command >. For tasks with dependencies (e.g. task Y in Fig. 1A and B) they are located at the output/ < seda_command_after > directory (where < seda_command_after > is the ID of each predecessor task, defined in the after property). Fourth, the output files of each task are located at the output/ < seda_command > directory. Finally, command parameters are taken from a file with the params/ < seda_command > . < extension > name (as Fig. 1C shows, the extension can be “.cliParams”, for standard CLI parameters, or “.sedaParams”, for SEDA configuration files in JSON format).

Since it is based on Compi, all the features provided by this framework (such as logging or partial execution) are also available. In addition to such functions, “SEDA pipelines with Compi” includes several extra features, specifically: (i) the support for running non-SEDA tasks (e.g. Bash scripts, as task Z in Fig. 1A), (ii) the ability to run commands in batches of a given size, essential for handling a large volume of input files or when resources are limited (by default, all the input files are processed in a single SEDA execution), and (iii) the generation of comprehensive and meaningful logs.

Also, as Fig. 1B shows for the task with id X_2, the input files can be provided as a plain text file at input/lists/ < seda_command > .txt. This is useful for re-analyzing only a subset of files such as new files not analyzed previously or files that failed at some point.

### Integration in GNU/Linux environments

The integration of SEDA in GNU/Linux environments was greatly improved in this new release. First, manual pages (or man pages) are now included in the GNU/Linux distributables and can be shown with man, as is usually done for most command-line programs. This complements the help provided by the own SEDA CLI application as well as the exhaustive online documentation [13]. The new SEDA release now offers installation packages for APT (Advanced Package Tool), used in Debian-based distributions like Ubuntu or Kubuntu, RPM (Red Hat Package Manager), used in Fedora or CentOS, among others, and Snap, a package manager available across a range of Linux distributions.

## Use cases

### Auto-phylo

Auto-Phylo is a pipeline maker tool for phylogenetic studies [14]. It has a variety of prebuilt modules for performing different tasks related to BLAST execution, FASTA file processing, alignment, tree building, model checking, gene annotation, detection of positively selected amino acid sites, and divergence estimates.

Users can combine each module at their convenience by following a simple syntax as long as they are compatible between them (i.e. the output of one module matches the required input of the next one), and the execution engine takes care of running the pipeline. The advantage of using Auto-Phylo is that it eases the creation of custom pipelines, eliminates human errors, and ensures reproducibility across operating systems and laboratories. This is a straightforward yet useful use case of the new SEDA CLI. All modules under the categories “Blast”, “FASTA file processing”, “Model checking”, “Gene annotation”, and “Divergence estimates”, as well as five out of nine modules under “Alignment”, and one out of 11 under”Tree building”, use SEDA-CLI operations. One of such modules, called “CGF_and_CGA_CDS_processing”, implements 12 SEDA CLI operations to process CDS FASTA files, downloaded from NCBI Assembly database for phylogenetic studies.

### Automated SEDA protocols

As noted before, the “SEDA pipelines with Compi” framework was used to automate three SEDA protocols for preparing datasets for large-scale phylogenetic analyses, obtaining protein family members, and performing phylogenomic studies.

The first protocol, available at GitHub [19], is designed to process a large number of coding sequence files to retrieve sequences showing similarity to a given gene. In other words, it allows preparing datasets for large-scale phylogenetic analyses. The provided test data include all configuration files for the GULO gene case study, which can be easily adapted to work with other genes.

The second protocol, available at GitHub [20], is aimed at retrieving all members of a given protein family using the PfamScan operation to annotate sequences. The provided test data include all configuration files for the case study of mucin proteins and can be easily adapted to other case studies.

Finally, the third protocol, available at GitHub [21], shows how to retrieve files and prepare datasets to be used in detailed phylogenomic studies. The test data provided focus on the use of mitochondrial genomes to pinpoint the most likely phylogenetic relationship between Rosaceae species. The protocol can be easily adapted to other species by simply changing the input data files.

## Conclusions

The new SEDA release featured here complements the previous GUI with a CLI and enhances its integration in GNU/Linux environments through the inclusion of man pages and new distributables. Both interfaces are readily accessible via the official Docker image, which is compatible with other container technologies, such as Singularity. In addition to introducing new functionalities, we have also presented a framework for easily constructing pipelines based on SEDA commands.

Through this update, our aim is to elevate SEDA from being a helpful GUI tool for researchers working with FASTA to becoming a fundamental part of pipelines and scripts for the analysis of this kind of data. Thanks to both the GUI and CLI interoperability and the “SEDA pipelines with Compi” framework, any researcher can easily design a protocol using the GUI (i.e. configuring and running each operation) and then build a distributable and reproducible pipeline that can be shared with other researchers.

## Availability and requirements

- Project name: SEDA (SEquence DAtaset builder)
- Project home page: http://www.sing-group.org/seda
- Archived version: 10.5281/zenodo.10201605
- Operating system(s): Linux, Windows and Mac OS.
- Programming language: Java 8
- Other requirements: Docker (optional) and/or third-party software like BLAST, Clustal Omega, etc. Check the manual for a complete list (http://www.sing-group.org/seda/manual/installation-and-configuration.html#dependencies-1).
- License: GNU GPL-3.0.
- Any restrictions to use by non-academics: e.g. licence needed

### Supplementary Information

Supplementary Material 1: SEDA operations. Table with all commands and operations in SEDA.

Supplementary Material 2: JSON configuration files of three different operations. JSON configuration files of three different operations (1. Remove isoforms; 2. BLAST; 3. Filtering).
