# Supplementary material for: SEDA 2024 update: enhancing the SEquence DAtaset builder for seamless integration into automated data analysis pipelines
Source: BMC Bioinformatics. 2024 May 27;25:200. doi: 10.1186/s12859-024-05818-2 (PMC11131258; doi:10.1186/s12859-024-05818-2)
Supplement: Supplementary file 1 — Supplementary Material 1: SEDA operations. Table with all commands and operations in SEDA. [file 12859_2024_5818_MOESM1_ESM.pdf]

Group	GUI Operation	CLI Command	Brief description
Alignment-related	Clustal Omega alignment	clustal-align	Use Clustal Omega to align the selected FASTA files.
	Concatenate sequences	concatenate	Concatenate sequences from selected files, according to FASTA header information, into a single output FASTA.
	Consensus sequence	consensus	Create a consensus sequence using the set of aligned sequences present in the selected FASTA file(s).
	Trim alignment	trim	Trim sequence alignments to remove alignment gap stretches at the beginning and end of the alignment.
	Undo alignment	Undo-alignment	Undo a sequence alignment by removing '-' from sequences.
BLAST	BLAST	blast	Perform BLAST queries using the selected FASTA files as a single or mutiple independent database(s).
	BLAST: two-way ortholog identification	blast-ortholog	Find sequence orthologs in a set of FASTA files using the Reciprocal Best Hits method.
	NCBI BLAST	blast-ncbi	Perform a BLAST query through the NCBI web server (https://blast.ncbi.nlm.nih.gov/Blast.cgi).
	UniProt BLAST	Blast-uniprot	Perform a BLAST query through the UniProt web server (https://www.uniprot.org/blast/)
Filtering	Base presence filtering	base-filtering	Filter sequences based on the percentages of their bases (nucleotides or amino acids).
	Filtering	filtering	Filter sequences based on different criteria (e.g. sequence length, non-multiple of three, or in-frame stop codons presence, among others).
	Pattern filtering	pattern-filtering	Filter sequences based on a text pattern that can be interpreted as either regular expression or plain text.
	Remove isoforms	remove-isoforms	Keep the isoform with the size closest to that specified, and remove all other isoforms, identified based on a shared word of specified length.
	Remove redundant sequences	remove-redundant	If several identical sequences (or subsequences, if chosen) are found, keep only one (user may choose to merge headers).
Gene Annotation	Augustus (SAPP)	augustus-sapp	Annotate an eukaryotic genome or sequence of interest by predicting genes using Augustus.
	Conserved Gene Annotation	cga	Obtain CDS annotations with CGA, using selected files and a FASTA file with the reference sequence.
	getorf (EMBOSS)	getorf	Find and extract all open reading frames (ORFs) with length longer than that specified by user, using the getorf program from the EMBOSS suite.
	ProSplign/ProCompart Pipeline	prosplign-procompart	Obtain CDS annotations with ProSplign/ProCompart, using selected files and a file with reference protein sequences.
	Splign/Compart Pipeline	splign-compart	Obtain CDS annotations with Splign/Compart, using selected files and a file with reference CDS sequences.
General	Compare	compare	Make all possible pairwise comparisons of selected files to find common and unique sequences.
	Grow sequences	grow	Grow sequences by merging those that show an overlap longer than that specified.
	Merge	merge	Merge all the selected input FASTA files into a single output FASTA.
	Regular expressions split	split-regex	Split each input FASTA file based on regular expression patterns.
	Remove stop codons	remove-stop-codons	Remove stop codons (TGA, TAG, and TAA) at the end of sequences in selected files.
	Reverse Complement	reverse-complement	Convert the sequences into the reverse, complement, or reverse complement counterparts.
	Split	split	Split each input FASTA file into several FASTA files (with the possibilty of random sequence sampling).
	Translate	translate	Translate nucleic acid sequences to their corresponding amino acid sequences.
Protein Annotation	PfamScan	pfamscan	Search and annotate sequences against the Pfam-A HMM library using the EMBL-EBI web service (https://www.ebi.ac.uk/Tools/pfa/pfamscan/)
Reformatting	Disambiguate sequence names	disambiguate	Disambiguate duplicated sequence identifiers, by adding a prefix, for instance.
	NCBI rename	rename-ncbi	Information from the NCBI Taxonomy Browser associated with a GCA/GCF code can be prefixed, sufixed or overwriten (can be applied to both sequence file names and sequence headers).
	Reallocate reference sequences	reallocate	Find one or more sequences (i.e. the reference sequences) using a pattern filtering option and reallocate them at the beginning of the file.
	Reformat file	reformat	Change the format of a FASTA file (sequence fragment length, line break type, and sequence case).
	Rename header	rename-header-add-word, rename-header-multipart, rename-header-replace-interval and rename-header-replace-word	Modify sequence headers by adding, deleting or replacing information.
	Sort	sort	Sort sequences by sequence length or sequence header (alphabetically).
