# Supplementary material for: SEDA 2024 update: enhancing the SEquence DAtaset builder for seamless integration into automated data analysis pipelines
Source: BMC Bioinformatics. 2024 May 27;25:200. doi: 10.1186/s12859-024-05818-2 (PMC11131258; doi:10.1186/s12859-024-05818-2)
Supplement: Supplementary file 2 — Supplementary Material 2: JSON configuration files of three different operations. JSON configuration files of three different operations (1. Remove isoforms; 2. BLAST; 3. Filtering). [file 12859_2024_5818_MOESM2_ESM.pdf]

1. Remove isoforms

```
{
   "removeIsoformsTransformationProvider" : {
      "minimumWordLengh" : 8,
      "defaultSequenceIsoformSelector" : {
         "referenceSize" : 10,
         "tieBreak" : "LONGEST"
      },
      "regexHeaderMatcher" : {
         "string" : "^[^_]*_[^_]*",
         "headerTarget" : "NAME",
         "regexConfig" : {
            "group" : 0,
            "quotePattern" : false,
            "caseSensitive" : false
         }
      },
      "sequenceHeadersJoiner" : {
         "target" : "NAME",
         "delimiter" : ", ",
         "prefix" : "[",
         "suffix" : "]"
      },
      "removedIsoformsFilesDirectory" : "/tmp/remove-isoforms"
   }
}
```

2. BLAST


```
{
   "blastTransformationProvider" : {
      "dockerBlastBinariesExecutor" : {
         "dockerImage" : "singgroup/seda-blast"
      },
      "storeDatabases" : true,
      "databasesDirectory" : "/tmp/blast/databases",
      "storeAlias" : true,
      "aliasFile" : "/tmp/blast/alias",
      "databaseQueryMode" : "EACH",
      "blastType" : "BLASTN",
      "queryFile" : "/tmp/blast/input/query",
      "eValue" : 0.05,
      "maxTargetSeqs" : 500000,
      "extractOnlyHitRegions" : false,
      "hitRegionsWindowSize" : 0
   }
}
```

3. Filtering


```
{
   "filteringConfigurationTransformationProvider" : {
      "startingCodons" : [ "ATG", "CAG" ],
      "headerFilteringConfiguration" : {
         "useFilter" : false,
         "mode" : "KEEP",
         "level" : "SEQUENCE",
         "min" : 0,
         "max" : 10,
         "filterType" : "SEQUENCE_NAME",
         "quotePattern" : false,
         "regexGroup" : 0,
         "caseSensitive" : false,
         "headerTarget" : "ALL"
      },
      "maxNumOfSequences" : 0,
      "maxSequenceLength" : 0,
      "minNumOfSequences" : 1,
      "minSequenceLength" : 0,
      "referenceIndex" : 1,
      "removeBySizeDifference" : true,
      "removeIfInFrameStopCodon" : true,
      "removeNonMultipleOfThree" : false,
      "sizeDifference" : 10
   }
}
```
